# Synthesis of Ag Nanoparticles-Decorated CNTs via Laser Ablation Method for the Enhancement the Photocatalytic Removal of Naphthalene from Water

**DOI:** 10.3390/nano11082142

**Published:** 2021-08-22

**Authors:** Fowzia S. Alamro, Ayman M. Mostafa, Khulood A. Abu Al-Ola, Hoda A. Ahmed, Arafat Toghan

**Affiliations:** 1 Department of Chemistry, College of Science, Princess Nourah bint Abdulrahman University, Riyadh 11671, Saudi Arabia; fsalamro@pnu.edu.sa; 2 Laser Technology Unit, Center of Excellent for Advanced Science, National Research Centre, 33 El Bohouth st. (Former El Tahrir st.), Dokki, Giza 12622, Egypt; 3 Spectroscopy Department, Physics Division, National Research Centre, 33 El Bohouth st. (Former El Tahrir st.), Dokki, Giza 12622, Egypt; 4 Center for Imaging and Microscopy (CIM), Zewail City of Science and Technology, October Gardens, 6th of October, Giza 12578, Egypt; 5 Chemistry Department, College of Sciences, Al-Madina Al-Munawarah, Taibah University, Al-Madina 30002, Saudi Arabia; Kabualola@taibahu.edu.sa; 6 Department of Chemistry, Faculty of Science, Cairo University, Cairo 12613, Egypt; ahoda@sci.cu.edu.eg; 7 Chemistry Department, College of Sciences, Yanbu, Taibah University, Yanbu 30799, Saudi Arabia; 8 Chemistry Department, Faculty of Science, South Valley University, Qena 83523, Egypt; arafat.toghan@yahoo.com; 9 Chemistry Department, College of Science, Imam Mohammad Ibn Saud Islamic University (IMSIU), Riyadh 11623, Saudi Arabia

**Keywords:** PLAL, laser ablation, CNT, nanocomposites, catalytic efficiency

## Abstract

Silver nanoparticles (Ag NPs) were decorated with different amounts on the exterior walls of carbon nanotubes (CNTs) by a laser ablation assisted method, especially in liquid media to be applied as a good adsorption material against naphthalene. The laser ablation time was controlled the amount of decoration Ag NPs on CNTs. The prepared nanocomposite was analyzed via different analytical techniques. Ag NPs with a small size distribution of 29 nm are uniformly decorated with spherical shape on CNTs walls. The disorder degree of tubular structure and shifting of the vibrational characteristic peaks increase with the increase in the decoration of Ag NPs. After that, the prepared samples were investigated for the removal of naphthalene. These studies of loading Ag NPs with different amounts on the surface of CNTs act as a promising material for water treatment.

## 1. Introduction

Water can be represented as a vital component of the world, and it is essential for our life. Water pollution is mostly caused by industrial and climate changes, which occurs when toxins are left without being treated or removed from the environment. Organic dyes, which presented in the industrial wastewater during the manufacturing of paper, cosmetics, and textiles, are major water contaminants. Organic dyes in water that contain benzene rings are harmful to health, which need to find a treatment process for contaminated wastewaters. There are numerous techniques for extracting contaminants and organic dyes from all types of water. From these methods, catalytic degradation of organic pollutants has been proven to be one of the most successful and cost-efficient strategies for dealing with environmental pollution [1,2,3,4,5].

Carbon nanotubes (CNTs) have captivated the attention since their discovery because of their unique structure and characteristics, which include a high active surface with respect to volume, excellent strength and flexibility, and chemical stability. Furthermore, they are also ideal support for nanosized metals and metal oxides that are embedded in the links to the CNTs’ exterior walls [6,7,8,9,10]. Besides, silver nanoparticles (Ag NPs) are a good additive material, and it is a valuable metal with a wide range of applications based on their promising properties. When Ag NPs employed as additives, they can significantly improve CNTs’ physicochemical properties, allowing them to be used and developed on different applications fields as sensors, catalytic degradation, optoelectronics, and biomedical applications [11,12,13,14]. The previous studies showed that the coating of nanotubes with nanoparticles could alter or justify some of their characteristics. The producing nanocomposites from these additions have been shown that Ag NPs/CNTs nanocomposites merit than CNTs or Ag NPs alone in various domains, such as hydrogen storage and catalysis [15,16,17,18,19].

Several methods for supporting different nanoparticles on CNTs have been invented as ball milling [20], photochemical reaction method [21], chemical reduction method [22]. The method of pulsed laser ablation in liquid environment (PLAL) was considered as the most versatile and promising way for fabricating metal or metal oxide-CNTs nanocomposites. PLAL method is one of the most significant, successful, and straightforward methods for producing metal oxide nanoparticles, that offers several benefits over other traditional physical and chemical techniques, including cleanliness, stability of the produced NPs colloids, ease of chemical preparation, and the lack of a vacuum chamber. This approach is the most versatile and powerful tool due to the capability for changing the particle shape/size by optimizing operational laser variables such as irradiation time, pulse duration, energy density, wavelength, and the ablation time [23,24,25,26,27,28].

In the previous work, the degradation efficiency resulted from Ag NPs and their decoration of CNTs against naphthalene showed that: In 2020, E. Fosso-Kankeu et al. succeeded in synthesis ZnO/Ag/GO nanocomposites by chemical method and degradation efficiency against naphthalene was studied, which was reached up to about 90% in less than an hour [29]. Also, S. Nasreen et al. prepared Ag NPs and Cu NPs via biological method by using different types of plants and reached efficiency more than 98% for degradation efficiency against naphthalene [30]. Therefore, it was assumed that decoration of CNTs with Ag NPs for catalytic applications is an excellent concept via simple one-step method of PLAL technique as a fast and eco-friendly method. It almost certainly performs better in catalytic reduction against different hazardous organic compounds [31,32].

Herein, we present a simple, one-step, and environmentally friendly technique in this study to decorate CNTs with Ag NPs with different amounts via laser assistant method based on generation of Ag NPs via the ablation of Ag plate immersed in solution from functionalized CNTs and increasing the amount of decoration via ablation time. The prepared nanocomposite performance is investigated by different characterization techniques as transmission electron microscopy, spectrophotometer, X-ray diffraction, Thermal gravimetric analysis, Fourier transform infrared and Raman spectroscopy, and atomic absorption spectroscopy. This research aims to develop an efficient method for synthesizing Ag NPs/CNTs nanocomposites with outstanding catalytic adsorption performance.

## 2. Materials and Experimental Works

### 2.1. Materials

Carbon nanotubes (CNTs) and silver plates were provided from Merck. Nitric acid (HNO_3_) was provided by Fischer Scientific, Leicester, UK.

### 2.2. Functionalization Process of CNTs

The functionalization procedure of the CNTs was prepared based on previous work [33]. It was based on mixing CNTs with acidic solvents—nitric acid and sulfuric acid in a ratio of 3:1 by volume—to create functional groups on their tubular outer surface. They act as active sites on the CNTs, allowing to catch the nanoparticles in the aqueous solution. The nanocomposite structure can be synthesized via the decoration of nanoparticles on the CNTs’ outer surface by the presence of these functional groups.

### 2.3. Preparation of Ag/CNTs Nanocomposite by PLAL

**Figure 1:**
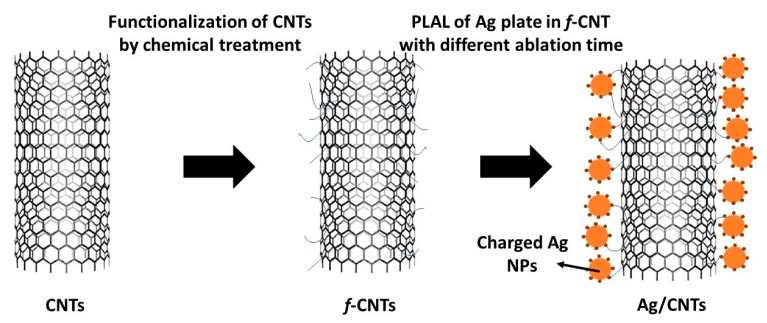
Schematic diagram of the used CNTs and their decoration by Ag NPs with different concentrations based on tuning laser ablation time.

The pulsed laser ablation method is based on using the nanosecond pulsed laser (Continuum laser, PL9000, Santa Clara, CA, USA) to generate laser energy =120 mJ, pulse duration = 7 ns, and laser wavelength = 1064 nm. This laser beam was focused and directed to the upper surface of the silver plate that was immersed in liquid solution (functionalized carbon nanotubes) by 7 cm of a plano-convex lens to form a laser beam with an effective spot size, which had a diameter of about 0.78 mm. The ablation time was used to change the generated amount of Ag NPs, which was directed to decorate the tubular’s surface of CNTs as shown in Figure 1. By PLAL method, the directed laser beam on the target surface in a very small cross-section produces high energetic laser pulse, followed by creating the multi-focused-photons, but in a very special condition. This condition is produced from inside a liquid media, allowing to produce laser induce plasma surrounded by liquid layer, leading to produce plasma-induced pressure and generating charged tiny particles (positively charges) approached to be in a nanoscale, which has a high capability to interact with functionalized materials (e.g., functionalized CNTs) suspended in the surrounding medium during ablation process. So, for the ablation of Ag plate immersed in liquid medium from functionalized CNTs, charged nanoparticles from Ag were generated and dispersed in the functionalized CNTs solution. The cationic charges on the outer surface of metal Ag NPs helped to be attracted by the anionic active sites on the CNTs’ tubular surface to form CNTs decorated with Ag NPs. Therefore, without the functionalization process of CNTs, the decoration process could not be accomplished [23].

### 2.4. Determination of Ag Concentration on the Prepared Nanocomposite

The cellulose filter paper was used as a substrate in a filtration system connected with a vacuum pump to collect CNTs with their decorated Ag NPs, and the solution containing the unloading Ag NPs was passed from the porous of filter paper. Then the CNTs paste were collected and washed with ultra-pure water to ensure that the remaining Ag NPs were attached to CNTs, followed by re-filtrating and drying at 50 °C. After that, the collected CNTs were washed with ultra-pure water to ensure that the remaining Ag NPs are attached to the CNTs, followed by re-filtrating and re-drying again. To estimate the total amount of decorated Ag nanoparticles in the CNTs, the prepared nanocomposite with different ablation times was immersed in acidic solution from 15 mL nitric acid at a concentration of 70%. Then, Ag^+^ concentration was calculated by AAS. According to Ag nanoparticles present only on the outer surface of the CNTs, the loading amount of Ag in CNTs ($W_{Ag}$) were calculated by the following expression [34,35]:

$$W_{Ag}=\frac{C_{Ag^{+}}\;X\;V}{M-C_{Ag^{+}}\;X\;V}$$

where $C_{Ag^{+}}$, *V*, and *M* are the concentration of Ag^+^ in the solution (measured from AAS), the volume of solution (250 mL), and the weight of Ag NPs/CNTs after completely drying (5 mg). The concentration of Ag nanoparticles corresponding to the different ablation times was determined by AAS (Table 1).

### 2.5. Determination of the Total Amount of Generated Ag NPs Using the PLAL Technique

The generation of Ag NPs via PLAL method was based on the ablation process of Ag plate, and all process was carried inside the closed system (vial or beaker) without any by-products, and the amount of Ag NPs was related to the weight loss in the main precursor (Ag plate). Therefore, the total amount of generated Ag NPs (loaded on the CNTs and free NPs) was determined by the following equation:

$$W_{\mathrm{total}\;\mathrm{Ag}\;\mathrm{NPs}}=W_{\mathrm{Ag}\;\mathrm{plate}\;\mathrm{before}\;\mathrm{PLAL}}-{\;W}_{\mathrm{Ag}\;\mathrm{plate}\;\mathrm{after}\;\mathrm{PLAL}}$$

where *W_total Ag NPs_*, *W_Ag plate before PLAL_*, and *W_Ag plate before PLAL_* are the total amount of generated silver nanoparticles during PLAL process, the weight of Ag plate before the ablation process, and the weight of Ag plate after the ablation process. By knowing the volume of the solution (10 mL), the concentration of generated Ag NPs can be simply calculated as tabulated in Table 1. These results are compatible with AAS analysis of the produced nanocomposite without a filtration process. The effect of nitric acid helped to collect all loaded and unloaded Ag NPs to be determined as total generated Ag NPs.

### 2.6. Investigation Techniques

The crystalline structure was obtained via an X-ray diffractometer (Shimadzu 7000, Tokyo, Japan). The qualitative and quantitative analyses were determined by Energy dispersive X-ray spectrometry, connected to a Field Emission-Scanning Electron Microscopy (Quanta FEG 250, FEI, Brno-Černovice, Czech Republic). The catalytic reduction performance was obtained by a UV-Visible spectrophotometer (JASCO, 570, Tokyo, Japan). The carbon nanotubes defect was carried out by the WITec alpha 300 R confocal Raman spectrometer. Chemical composition was carried out by Fourier-transform infrared spectroscopy (JASCO 6100 spectrometer, Tokyo, Japan). The thermal combustion analysis was carried out by the Perkin Elmer TGA thermogravimetric Analyzer. The concentration of Ag NPs was detected by atomic absorption spectroscopy (Perkin–Elmer AAnalyst 100, Waltham, MA, USA).

### 2.7. Catalytic Degradation Application Study

The studied adsorbent materials were added at a concentration of 0.2 g/L to the 40 mg/L naphthalene aqueous solution at room temperature (37 °C) with stirring for about 10 min in the dark before being exposed to light to make sure that the adsorbent surfaces and naphthalene molecules have interacted. UV–vis absorption spectra were used to determine the residual naphthalene content in the water. Furthermore, the absence of naphthalene smell from the solution showed that it was removed from the solution. The photocatalytic activities were evaluated in a beaker with a magnetic stirrer under UV light of 16 W. Samples were taken at regular intervals to evaluate the reaction via a photocatalyst to eliminate naphthalene. The degradation efficiency of naphthalene was measured using a spectrophotometer.

## 3. Results and Discussion

### 3.1. Study of Nanocomposite

**Figure 2:**
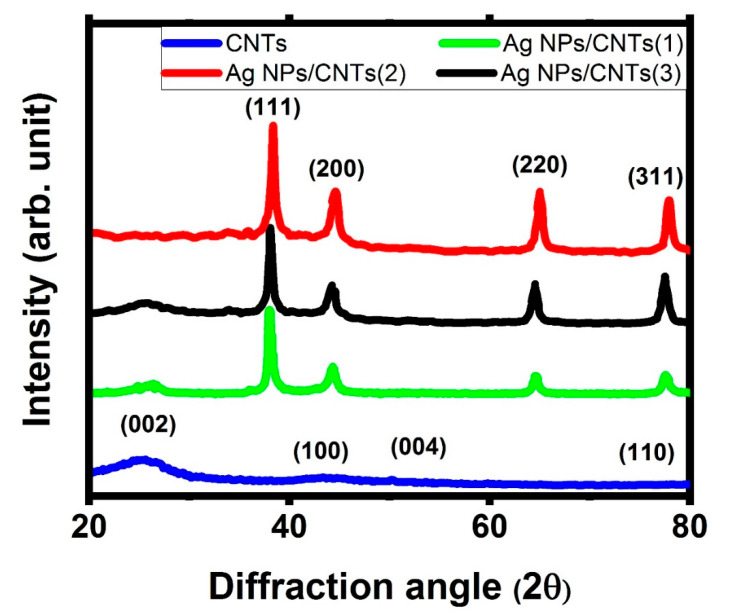
XRD diffractogram of the used CNTs and their decoration by Ag NPs with different concentrations based on tuning laser ablation time.

Figure 2 shows the XRD patterns of CNTs and their nanocomposite with different amounts of Ag NPs, done by varying ablation time. For CNTs, the characteristic peaks at 25.29°, 43.41°, 52.11°, and 76.06° were responsible for the lattice plane of (0 0 2), (1 0 0), (0 0 4), and (1 1 0), respectively, based on JCPDS No. 75-1621 [10], while for ablation of Ag plate immersed in liquid media from functionalized CNTs, there are new characteristic peaks appeared at 38.17°, 44.27°, 64.55°, and 77.55° were responsible for the lattice plane of (1 1 1), (2 0 0), (2 2 0), and (3 1 1), respectively, which is based on JCPDS No.04-0783 [36]. These results proved that the effect of the ablation of Ag plate in *f*-CNTs solution leads to producing cubic structure of Ag NPs on the graphite structure of CNTs. Also, the intensity of the main crystalline peak (1 1 1) indicated an increase in the amount of Ag NPs decorated the tubular surface of CNTs. This behavior could be detected by focusing on the intensity of the main characteristic interplanar indices of (1 1 1) in comparison with the main characteristic peak of CNTs, which was related to the interplanar indices of (0 0 2). So, as the ablation time increases, the amount of decoration on CNTs’ surface with Ag NPs increases. Furthermore, the determination and the changes of the particle sizes of Ag NPs could be detected by the Scherrer equation [37,38].

D = (kλ)/(βcosθ)(1)

where β, θ, k, and λ are the broadening of the selective pattern line, the diffraction angle, the shape factor, and the exited X-ray laser source. For that, the average crystallinity size of (1 1 1) plane was about 29 nm for all prepared nanocomposites. This measurement confirmed that the ablation time did not make a valuable change on the particle size of Ag NPs, but it made a significant effect on the amounts of generated Ag NPs. That was related to that the ablation time is mainly affected on the generation of extra nanoparticles without any remarkable change in the generated nanoparticles.

**Figure 3:**
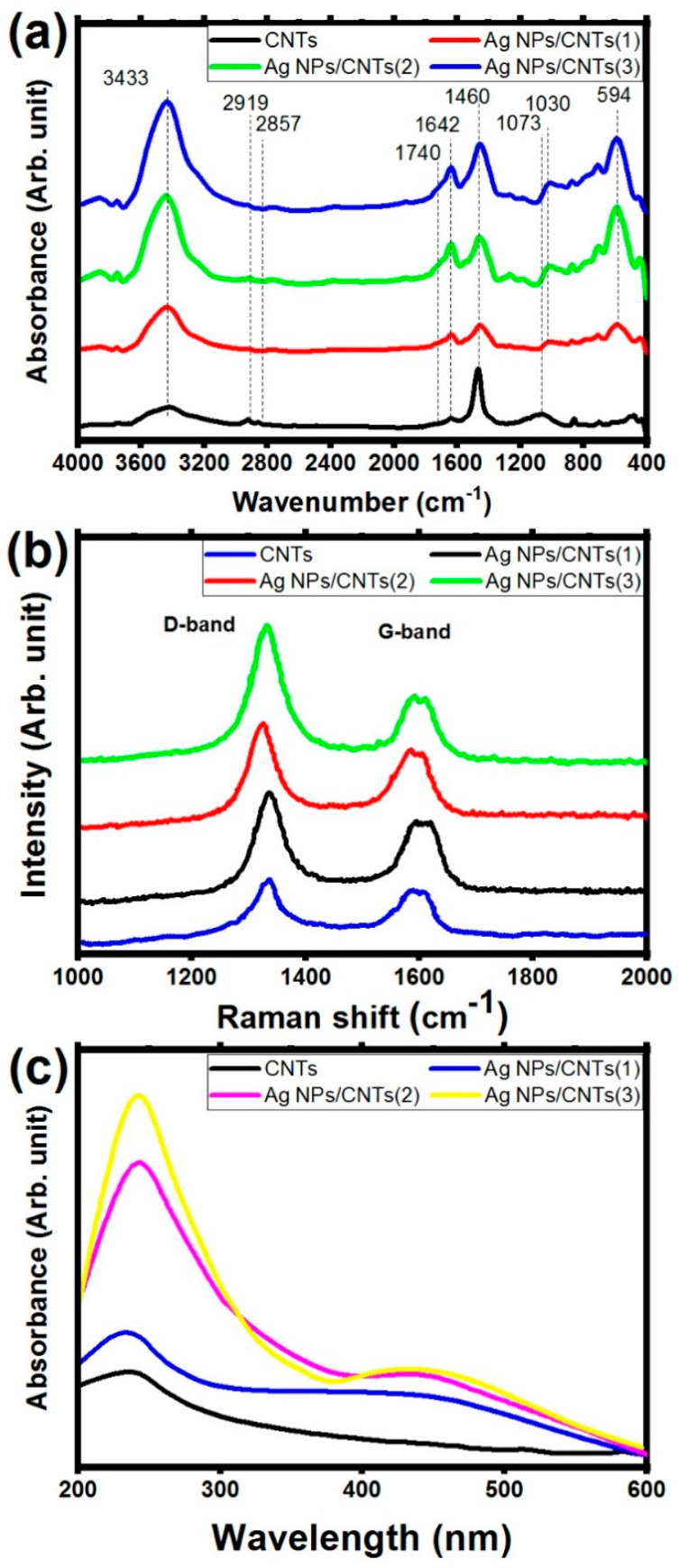
(**a**) FT–IR spectra, (**b**) Raman spectroscopy, and (**c**) UV-visible spectroscopy of the used CNTs and their decoration by Ag NPs with different concentrations based on tuning laser ablation time.

FT-IR and Raman studies were used to investigate the interaction between Ag NPs and functional groups on the outer surface of functionalized CNTs. Figure 3a showed that FT-IR spectra of the utilized CNTs and their nanocomposites with different amounts of Ag nanoparticles clearing that in the case of CNTs, it can be noticed that the existence of adsorption bands at 3433 cm^−1^ is corresponding to –OH stretching vibration. Also, the main characteristic peak of the functionalized carbon nanotube skeleton structure of CNTs was observed around 1642 cm^−1^ related to the functional group of C=C bonding of aromatic rings. The presence of the carbonyl functional group in the carboxylic functional group (-COOH) was detected around 1700 cm^−1^, confirming the effect of functionalization on the CNTs’ tubular outer surface. Besides, the presence of the stretching vibrational motion at 920 cm^−1^ was related to C-O functional group of carboxylic acid. These FT-IR data indicated that the oxidation treatment process was successfully produced on the outer surface of CNTs by adding functional carboxyl and hydroxyl groups [39,40,41,42]. In the case of decoration with Ag NPs, the interactions between silver ions and the hydroxyl group of CNTs are responsible for the change in the intensity and in the positions of the peaks in the range from 1400 cm^−1^ to 1000 cm^−1^. Besides, the presence of the vibrating starching modes C-Ag functional group at 594 cm^−1^. These data could represent a confirmation on the interaction. [43,44].

The crystallinity and structural changes of the carbon framework of CNTs before and after decoration with Ag NPs were investigated using Raman spectroscopy as shown in Figure 3b. From these spectra, CNTs sample has strong peaks around 1338 cm^−1^ (D band) and 1583 cm^−1^ (G band), whereas Ag/CNTs nanocomposite sample has equivalent peaks at 1322 cm^−1^ and 1594 cm^−1^. D band represents edges, various defects, and disordered in the carbonic structure from the vibration of sp^3^ hybridization from CNTs skeleton and impurities, whereas G band comes from the zone mode in CNTs structure, assigning to the ordered sp^2^ hybridization of C atoms in CNTs structure [45,46]. Compared to CNTs, Ag NPs/CNTs nanocomposites have a considerable frequency shift toward a lower D-band wavenumber (approximately 30 cm^−1^) due to the partial reduction in CNTs by decorating with Ag NPs on their external tubular surface, this result reveals a high level of disorder in the graphene layers of CNTs structure, as well as an increase in the number of defects, confirming the interaction between the external surface of CNTs structure and Ag NPs. Furthermore, when Ag NPs decorate CNTs structure, the intensity ratio of D band to G band (I_D_/I_G_) increases. That was related to increasing the degree of disorder due to the decoration of CNTs with Ag NPs [47,48,49,50]. The values of this ratio were approximately 1.19, 1.41, 1.46, and 2.22 for CNTs, Ag NPs/CNTs (1), Ag NPs/CNTs (2), and Ag NPs/CNTs (3) nanocomposites, respectively.

Figure 3c depicts the absorption properties of our produced samples. The CNT has a distinct peak at 240 nm, which could be attributed to the aromatic C–C bonds of the π–π* transitions. This peak was linked to a little perturbation in the wavelength region of 200 nm to 250 nm. The Ag/CNTs nanocomposite sample has a spectrum that is comparable to that of CNTs, with the primary absorption peak of Ag/CNTs at 240 nm. The absorption peak of the Ag NPs/CNTs nanocomposite sample became weaker and narrower than the absorption peak of the CNTs sample, which can be attributed to enhanced scattering of shorter wavelengths, notably maybe by the presence of the crystallite structure of the Ag nanoparticles. In addition, there is a new addition around the 450 nm peak, which can be attributed to the presence of plasmonic structure from the noble metal (Ag NPs). The intensity of this characteristic peak increases as the ablation time increases, in relation to the increase in amounts of decoration of Ag NPs on the surface on the CNTs [51,52]. Furthermore, the following equation may be used to compute the energy bandgap of the prepared samples [53]:

Eg (eV)= hcλ=1240λ

where *h*, *c*, and *λ* are the constant of plank, light speed, and the maximum absorption of the studied samples. From this equation, the optical energy bandgap is found to be equal 2.95 eV, 2.88 eV, and 2.86 eV for Ag NPs/CNT (1), Ag NPs/CNT (2), and Ag NPs/CNT (3), respectively.

**Figure 4:**
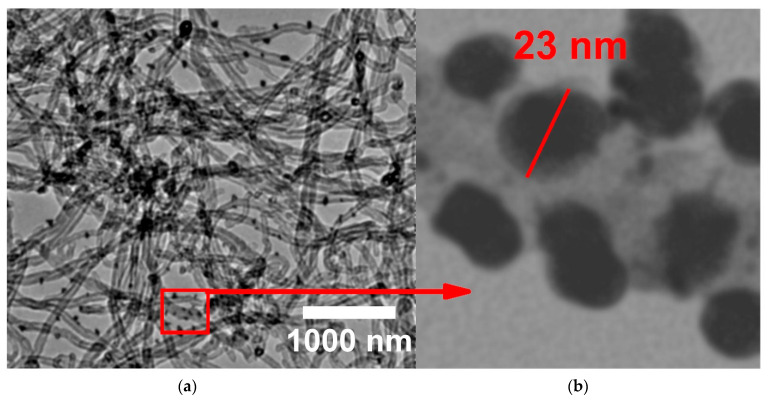
TEM image of the prepared Ag NPs/CNTs (1) nanocomposite formed via pulsed laser ablation of the silver plate for about 10 min (**a**) at low magnification (**b**) high magnification.

The morphology of Ag NPs/CNTs (1) nanocomposite structure was studied using TEM, as shown in Figure 4. CNTs had a smooth surface with diameter around 34 nm, whereas Ag NPs has a spherical form with a diameter of 23 nm, as seen in this image. Furthermore, decoration of Ag NPs on the external surface of CNTs has no effect on the structure of Ag NPs, and CNTs are evenly dispersed throughout the composite.

**Figure 5:**
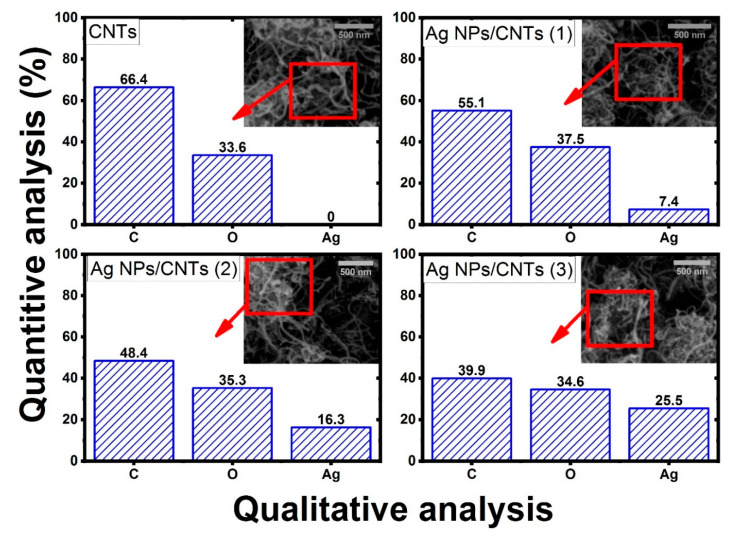
The SEM images and elemental analysis of the functionalized CNTs and their decoration by Ag NPs with different concentration based on tuning laser ablation time.

As shown in Figure 5, the energy dispersive of X-ray spectroscopic analysis was used to identify the chemical compositions of the prepared nanocomposite structure. The elemental analysis was studied on the selected area (1 μm × 1 μm) at the scale bar 500 nm via scanning electron microscope. In the case of CNTs, the consistent elements are only C and O, while in the case of the decoration of Ag NPs in functionalized CNTs, the produced spectrum shows the characteristic elements from Ag, C and O with different percentages based on the time of ablation. The percentage changes of these constituent elements were observed in that figure. From this study, it was cleared that, as the ablation time increases the amount of Ag increases and the amounts of C and O decreases. Also, there is no appearance of other elements rather than the main structure of CNTs and Ag elements.

As shown in Figure 5, the energy dispersive of X-ray spectroscopic analysis was used to identify the chemical compositions of the prepared nanocomposite structure. The elemental analysis was studied on the selected area (1 μm × 1 μm) at the scale bar 500 nm via scanning electron microscope. In the case of CNTs, the consistent elements are only C and O, while in the case of the decoration of Ag NPs in functionalized CNTs, the produced spectrum shows the characteristic elements from Ag, C and O with different percentages based on the time of ablation. The percentage changes of these constituent elements were observed in that figure. From this study, it was cleared that, as the ablation time increases the amount of Ag increases and the amounts of C and O decreases. Also, there is no appearance of other elements rather than the main structure of CNTs and Ag elements.

**Figure 6:**
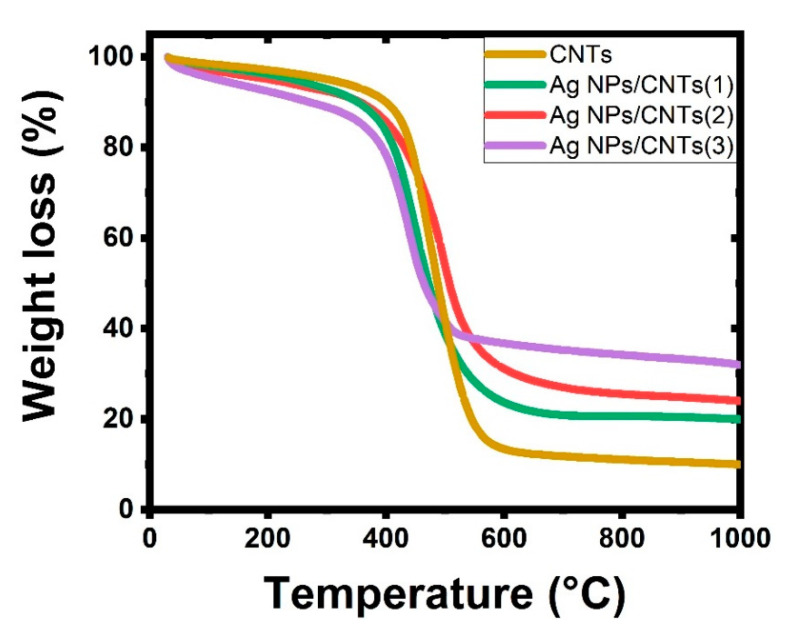
TGA of the used CNTs and their decoration by Ag NPs with different concentrations based on tuning laser ablation time.

Figure 6 shows the TGA curves of CNTs and their decoration with various amounts of Ag nanoparticles. The significant mass loss in the CNTs curve from 580 °C to 710 °C is related to the carbon structure of the CNTs decomposing. In the region up to 500 °C, there is a gradual and moderate mass loss of about 4% due to the elimination of oxygen functionalities generated during the functionalization process. CNTs almost burn out around 800 °C, with very few remaining weights. The remaining amount of mass loss at temperatures higher than 800 °C was related to the presence of the decorated Ag NPs. The overall mass loss of Ag/CNTs is reduced by up to 85%, 68%, and 50% till 800 °C, respectively, corresponding to varied ablation times in minutes (10, 20, and 30). The loading percentages of Ag NPs were related to the ablation time (25%, 37%, and 40%) [54,55,56,57].

The sample from the ablation of the Ag plate in *f*-CNTs is Ag NPs and CNTs in the form of CNT decorated by Ag NPs. Confirmation of the presence of CNTs and Ag NPs in the prepared nanocomposite was reveled via XRD analysis, UV-VIS absorption study, TGA analysis, and EDX analysis, while the decoration CNTs by Ag NPs was confirmed by FT-IR, Raman, and TEM.

From XRD analysis, the synthesized colloidal solution nanocomposites from the ablation of Ag plate immersed in *f*-CNTs consisted of only graphite structure of CNTs and metal Ag NPs in the cubic phase. Also, the effect of ablation time did not make any remarkable changes in the particle size of the prepared Ag NPs, but increase in the ratio amounts between Ag NPs with respect to CNTs structure. From UV-VIS absorption study, there is a new additional peak appeared in visible region, which can be related to the presence of plasmonic structure from noble metal (Ag NPs). Also, their intensity increases as the ablation time increases, related to the increase in the amounts of Ag NPs with respect to CNTs. From EDX analysis, the chemical identification on the prepared samples showed the presence of the characteristic elements from Ag, C, and O with different percentage for the decoration of Ag on *f*-CNTs, while Ag elements disappeared before starting the ablation process. From TGA analysis, the interaction between them was confirmed by the remaining amount of the mass loss at higher than 800 °C, which was related to the presence of the ablated Ag NPs.

From FT-IR analysis, there are slightly shift in the functional groups in the range from 1400 cm^−1^ to 1000 cm^−1^ related to the interactions between silver ions and the hydroxyl group presence on *f*-CNTs’ outer surface. Besides, the appearing of the fingerprint peaks of C-Ag functional group confirmed the interaction between them. So, this analysis could be represented as the first prove for the interaction between Ag NPs and CNTs. From Raman analysis, the increase in the number of defects in the structure of CNTs, confirming the interaction between the external surface of CNTs structure and Ag NPs. So, this analysis could be represented as the second prove for the interaction between Ag NPs and CNTs. From TEM image, Ag NPs successfully decorated the external surface of CNTs without making any effect on the structure of Ag NPs. So, this analysis could be represented as the third prove for the interaction between Ag NPs and CNTs.

### 3.2. Catalytic Activity

**Figure 7:**
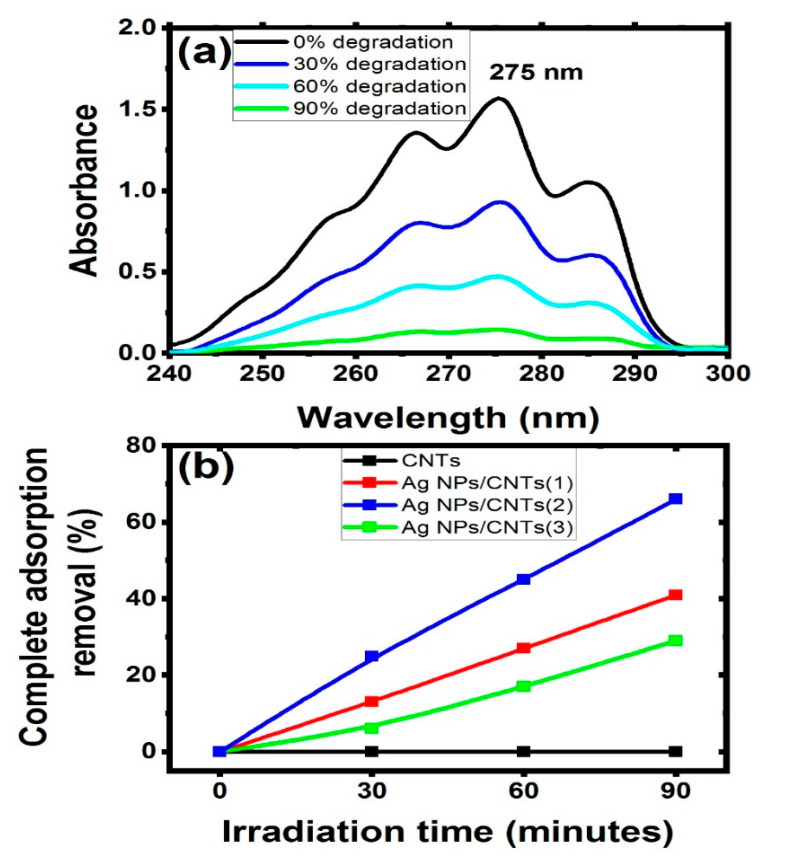
(**a**) Photocatalytic degradation of naphthalene by using Ag NPs/CNT(2), and (**b**) the relationship between irradiation time against the complete adsorption removal percentage of the used CNTs and their decoration by Ag NPs with different concentrations based on tuning laser ablation time.

The adsorption performance of the studied nanocomposite with different amount of silver were examined regarding to CNTs for the removal of naphthalene structure under light irradiation at room temperature (37 °C). The degradation efficiency of the studied samples against naphthalene is depicted in Figure 7a. In this figure, UV-visible investigation showed the main characteristic transition peaks of naphthalene, which was appeared at 275 nm [29,58]. After that, the prepared mixed solutions after adding the tested samples were irradiated, followed by studying the changes of the naphthalene spectra. Also, the effect of irradiation time on the mixed solution was produced with different amount for complete adsorption removal as shown in Figure 7b. The following equation was used to compute the photocatalytic degradation efficiency of naphthalene [59]:

$$Degradation\;effeciecny\;\left(\%\right)\;=\;\;\left(\left(A_{f}-A_{i}\right)/A_{i}\right)*100$$

where *A_f_* and *A_i_* are the values of naphthalene absorbance after and before irradiation at t time. From this figure, it was cleared that CNTs did not make any change in the degradation of naphthalene, while as Ag NPs started to be presented in the structure as a decorated material, the concentration of naphthalene started to be degraded. If Ag NPs exceed certain amount of decoration with respect to the number of CNTs, the efficiency of adsorption performance started to be decreased due to CNTs became brittle if holding more than certain amount [60].

**Figure 8:**
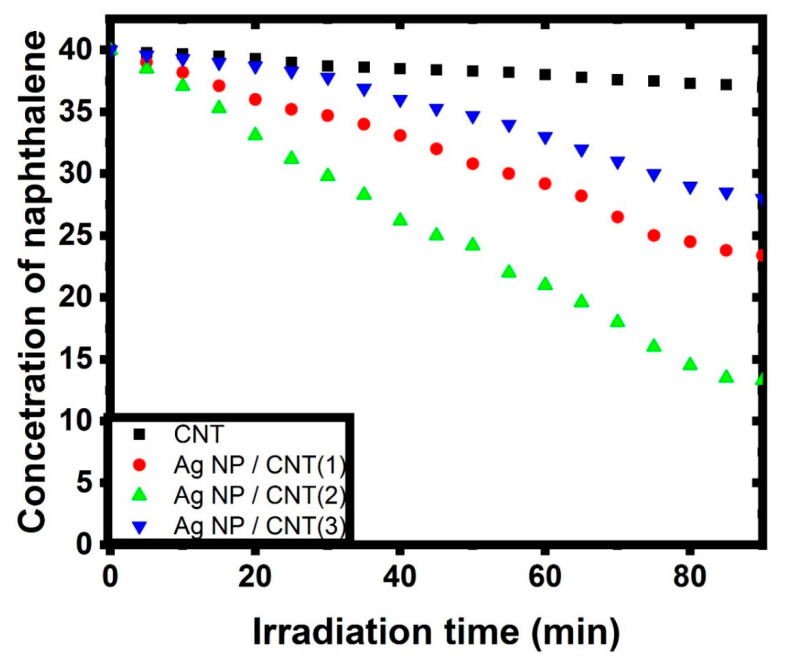
Decrease in naphthalene concentration with respect to different irradiations for CNTs and their decoration with different amounts of Ag NPs.

Furthermore, this figure was used to create a kinetic model for total adsorption and photocatalytic degradation of naphthalene in water solutions under irradiation light, which were investigated synchronously in the same experimental situations by varying the concentrations. The impact of initial naphthalene concentrations on adsorption/photodegradation rate by employing CNTs and their decorating with various amounts of Ag NPs can be seen in the decrease of naphthalene concentration during irradiation time (as seen in Figure 8). Furthermore, the reaction was almost complete for each starting concentration of naphthalene utilizing CNTs and their decorating samples with Ag NPs. After 80 min of light irradiation, the remaining concentrations of CNTs, Ag NPs/CNTs (1), Ag NPs/CNTs (2), and Ag NPs/CNTs (3) were found to be 36.6 mg/L, 31.6 mg/L, 10.7 mg/L, and 26.6 mg/L, respectively.

The presence of two processes (adsorption and photocatalytic) for the degradation of naphthalene may be simultaneously occurred and investigated using first-order, second-order, or Langmuir-Hinshelwood kinetic models [61]. The first process described in this reaction was related to the adsorption of naphthalene demonstrating that naphthalene absorption happens as a result of a process defined by the first order kinetic constant *k*_1_, which is given by the equation:

$$\ln\frac{C_{i}-C_{t}}{C_{t}}\;=\;-k_{1}t$$

**Figure 9:**
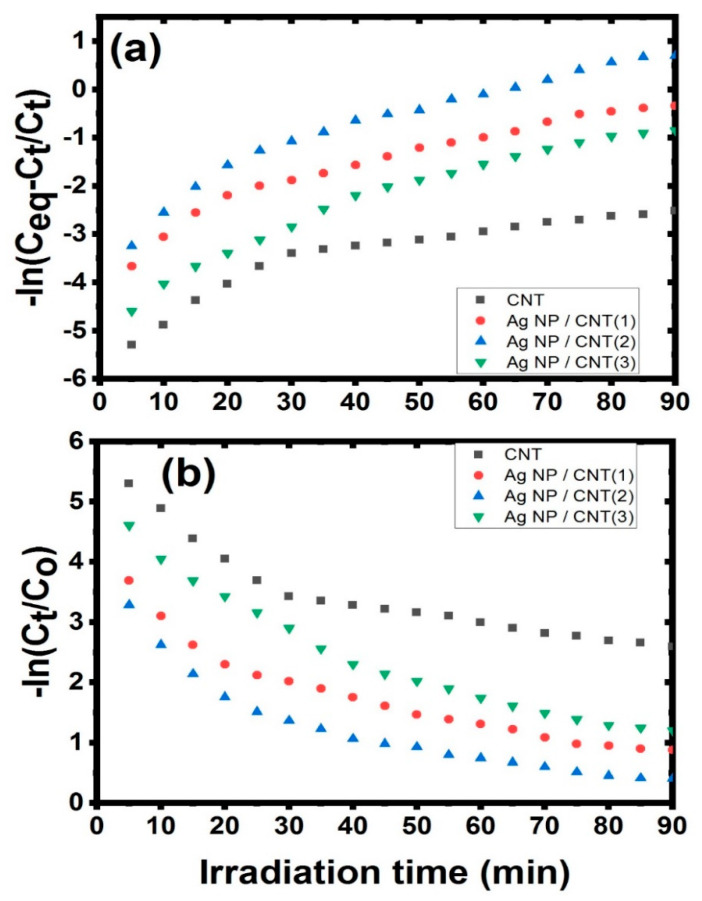
(**a**,**b**): Different kinetic models for CNTs and their decoration with different amount from Ag NPs.

While the second process described in this reaction was related to the photocatalytic degradation of naphthalene. The rate of photodegradation of naphthalene is characterized by the first order kinetic constant *k*_2_, which is given by the equation:

$$\ln\frac{C_{t}}{C_{i}}\;=\;-k_{2}t$$

where $C_{i},\;C_{t},\;and\;C_{eq}$ are the concentration of naphthalene at the beginning, at any time during the reaction process, and at equilibrium, respectively [62]. Figure 9 represented the kinetic modeling of the prepared samples against naphthalene. The calculation of the kinetic constant was tabulated in Table 2. From this data, the adsorption stage is limited by the sluggish in the photodegradation step. So, the adsorption behavior has a significant impact on the whole process [61].

**Figure 10:**
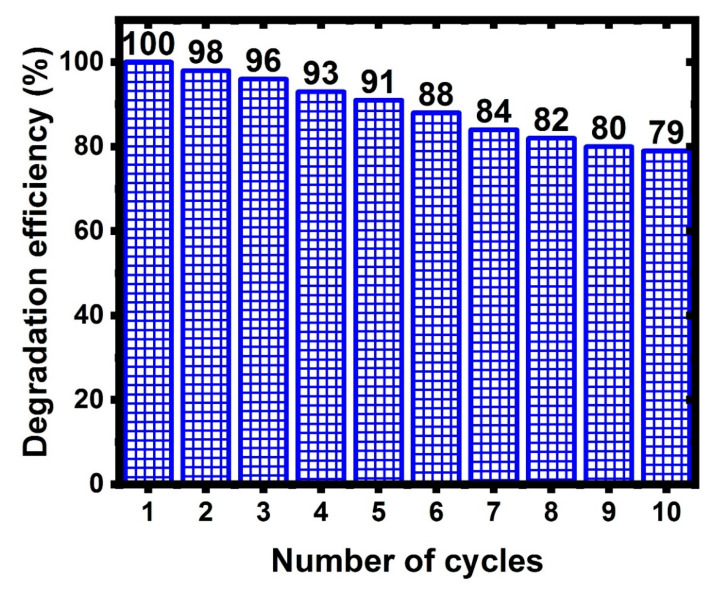
10 Cyclic time for using Ag NPs/CNT (2) nanocomposite for photocatalytic degradation.

Figure 10 shows the photocatalytic degradation of naphthalene, and the efficiency and durability of Ag NP/CNTs (2) as a reusable catalyst. Once the nanocomposite catalyst was prepared and utilized for the first degradation procedure, it was collected, recovered, and reused up to 10 times for photocatalytic degradation tests. From this figure, the efficiency of 66% degradation from naphthalene in 90 min was lowered from 100% to 79%. This result shows that the nanocomposite material has a high level of stability. However, the decrease in efficiency by about 21% after the tenth reuse was due to the nanocomposite being collected and washed by ultra-pure water before being reused.

### 3.3. Mechanism of Catalytic Performance

**Figure 11:**
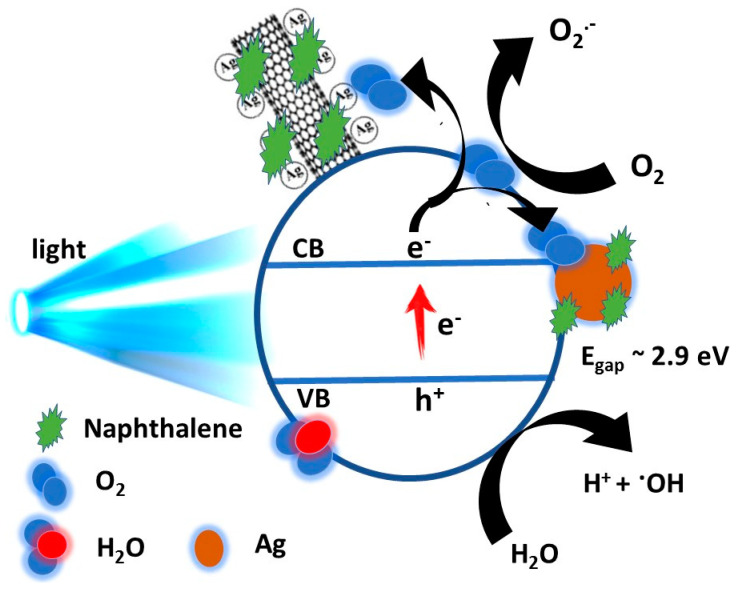
Schematic diagram of the photocatalytic degradation of naphthalene by Ag NPs/CNT.

The influence of visible light irradiation and the addition of Ag NPs/CNT were concluded to degrade naphthalene by the photocatalytic process. The oxidation of CNTs provides numerous functional groups—such as hydroxyl, carboxyl, and carbonyl—on the tubular surface of CNTs, as discussed before. These functional groups serve as active locations assisting CNTs in adsorbing Ag NPs to form the Ag NPs/CNT nanocomposite. Then, analogous to semiconductor–metal junctions, the Ag NPs/CNTs nanocomposites are built, and Ag NPs acts as electron acceptors [63], as mentioned in the schematic diagram of Figure 11.

Once the prepared nanocomposite material is inserted into the naphthalene solution, a number of interactions were carried out. Due to the obvious significant coupling between CNTs and naphthalene, a substantial number of naphthalene molecules are adsorbed on the tubular surface of the CNTs. The excited state of naphthalene molecules can be triggered by visible light irradiation. The electrons move from the exciting form of naphthalene to CNTs because of the high electron affinity of CNTs. CNTs allow electrons to freely travel without being scattered by any external defects. When moving electrons collide with the present locations of Ag NPs, they get trapped. The adsorbed oxygen atoms are transformed to superoxide anion radical ($O_{2}{}^{-}$) and hydroxyl radical ($OH^{.}$) by the electrons collected on the Ag NPs. As a result, these active oxygen species degrade naphthalene [64,65].

## 4. Conclusions

In this work, we used laser ablation to demonstrate a straightforward approach for producing Ag-decorated CNTs nanocomposites with different amounts based on tuning the laser ablation time to be applicable for water treatment from hazard organic compounds such as naphthalene. A nanocomposite structure was created via laser ablation method by making an Ag plate containing *f*-CNTs inside liquid media. The characterization methods showed that the vibration characteristic peaks of CNTs shifted due to the interaction of silver with the graphite layers of CNTs. The qualitative analysis did not show any element other than C, O, and Ag. The diffractogram spectra showed the presence of interplanar indices of the graphite and silver structures without any change in their crystallinity. The degree of disorder rises on increasing the decoration of Ag NPs, and the remaining amount of mass loss at a temperature higher than 800 °C due to the presence of the decorated Ag NPs on the CNTs’ surface.

## Figures and Tables

**Table 1:** Calculation of total concentration of Ag for the different prepared nanocomposites by AAS.

Sample Name	Laser Ablation Time (Minutes)	Total Concentration of Generated Ag NPs	Concentration of Ag NPs in the Prepared Nanocomposite
**CNTs**	0	0 μg/L	0 μg/L
**Ag/CNTs (1)**	10	7.3 μg/L	6 μg/L
**Ag/CNTs (2)**	20	15.2 μg/L	12 μg/L
**Ag/CNTs (3)**	40	33.5 μg/L	26 μg/L

**Table 2:** The kinetic constants (*k*_1_) and (*k*_2_) for the prepared samples.

	*k*_1_	*k*_2_
**CNT**	0.6	1.9 × 10^−2^
**Ag NPs/CNT (1)**	1	5 × 10^−2^
**Ag NPs/CNT (2)**	1.1	6.2 × 10^−2^
**Ag NPs/CNT (3)**	0.7	4 × 10^−2^

## Data Availability

The data presented in this study are available on request from the corresponding author.

## References

[1] Mostafa A.M., Mwafy E.A. (2020). Synthesis of ZnO/CdO thin film for catalytic degradation of 4-nitrophenol. J. Mol. Struct..

[2] Mostafa A.M., Mwafy E.A. (2020). The effect of laser fluence for enhancing the antibacterial activity of NiO nanoparticles by pulsed laser ablation in liquid media. Environ. Nanotechnol. Monit. Manag..

[3] Mwafy E.A., Mostafa A.M. (2020). Tailored MWCNTs/SnO_2_ decorated cellulose nanofiber adsorbent for the removal of Cu (II) from waste water. Radiat. Phys. Chem..

[4] Mostafa A.M., Mwafy E.A., Awwad N.S., Ibrahium H.A. (2021). Catalytic activity of Ag nanoparticles and Au/Ag nanocomposite prepared by pulsed laser ablation technique against 4-nitrophenol for environmental applications. J. Mater. Sci. Mater. Electron..

[5] Mostafa A.M., Mwafy E.A. (2020). Effect of dual-beam laser radiation for synthetic SnO_2_/Au nanoalloy for antibacterial activity. J. Mol. Struct..

[6] Khairy M., Naguib E.M., Mohamed M.M. (2020). Enhancement of Photocatalytic and Sonophotocatalytic Degradation of 4-nitrophenol by ZnO/Graphene Oxide and ZnO/Carbon Nanotube Nanocomposites. J. Photochem. Photobiol. A Chem..

[7] Mostafa A.M., Mwafy E.A., Awwad N.S., Ibrahium H.A. (2021). Synthesis of multi-walled carbon nanotubes decorated with silver metallic nanoparticles as a catalytic degradable material via pulsed laser ablation in liquid media. Colloids Surf. A Physicochem. Eng. Asp..

[8] Mostafa A.M., Mwafy E.A., Toghan A. (2021). ZnO nanoparticles decorated carbon nanotubes via pulsed laser ablation method for degradation of methylene blue dyes. Colloids Surf. A Physicochem. Eng. Asp..

[9] Abozied A.M., Mostafa A.M., Abouelsayed A., Hassan A.F., Ramadan A.A., Al-Ashkar E.A., Anis B. (2021). Preparation, characterization, and nonlinear optical properties of graphene oxide thin film doped with low chirality metallic SWCNTs. J. Mater. Sci. Technol..

[10] Mwafy E.A., Mostafa A.M., Awwad N.S., Ibrahium H.A. (2021). Catalytic activity of multi-walled carbon nanotubes decorated with tungsten trioxides nanoparticles against 4-nitrophenol. J. Phys. Chem. Solids.

[11] Gao L., Zhou F., Chen Q., Duan G. (2018). Generation of Pd@ Ni-CNTs from Polyethylene Wastes and Their Application in the Electrochemical Hydrogen Evolution Reaction. ChemistrySelect.

[12] Zannotti M., Vicomandi V., Rossi A., Minicucci M., Ferraro S., Petetta L., Giovannetti R. (2020). Tuning of hydrogen peroxide etching during the synthesis of silver nanoparticles. An application of triangular nanoplates as plasmon sensors for Hg^2+^ in aqueous solution. J. Mol. Liq..

[13] Mwafy E.A., Mostafa A.M. (2019). Multi walled carbon nanotube decorated cadmium oxide nanoparticles via pulsed laser ablation in liquid media. Opt. Laser Technol..

[14] Sarina S., Waclawik E.R., Zhu H. (2013). Photocatalysis on supported gold and silver nanoparticles under ultraviolet and visible light irradiation. Green Chem..

[15] Mwafy E.A., Mostafa A.M. (2020). Efficient removal of Cu (II) by SnO2/MWCNTs nanocomposite by pulsed laser ablation method. Nano-Struct. Nano-Objects.

[16] Naik S.S., Lee S.J., Theerthagiri J., Yu Y., Choi M.Y. (2021). Rapid and highly selective electrochemical sensor based on ZnS/Au-decorated f-multi-walled carbon nanotube nanocomposites produced via pulsed laser technique for detection of toxic nitro compounds. J. Hazard. Mater..

[17] Mwafy E.A., Gaafar M.S., Mostafa A.M., Marzouk S.Y., Mahmoud I.S. (2021). Novel laser-assisted method for synthesis of SnO_2_/MWCNTs nanocomposite for water treatment from Cu (II). Diamond Relat. Mater..

[18] Yu B., Chen Y. (2020). Conductive WO3-x@ CNT networks for efficient Li-S batteries. IOP Conference Series: Materials Science and Engineering.

[19] Velmurugan S., Palanisamy S., Yang T.C., Gochoo M., Chen S.-W. (2020). Ultrasonic assisted functionalization of MWCNT and synergistic electrocatalytic effect of nano-hydroxyapatite incorporated MWCNT-chitosan scaffolds for sensing of nitrofurantoin. Ultrason. Sonochem..

[20] Ma P.C., Tang B.Z., Kim J.-K. (2008). Effect of CNT decoration with silver nanoparticles on electrical conductivity of CNT-polymer composites. Carbon.

[21] Dinh N.X., Quy N.V., Huy T.Q., Le A.-T.J.J.o.N. (2015). Decoration of silver nanoparticles on multiwalled carbon nanotubes: Antibacterial mechanism and ultrastructural analysis. J. Nanomater..

[22] Ahmadpoor F., Zebarjad S.M., Janghorban K. (2013). Decoration of multi-walled carbon nanotubes with silver nanoparticles and investigation on its colloid stability. Mater. Chem. Phys..

[23] ElFaham M.M., Okil M., Mostafa A.M. (2020). Fabrication of magnesium metallic nanoparticles by liquid-assisted laser ablation. JOSA B.

[24] Mwafy E.A., Hasanin M.S., Mostafa A.M. (2019). Cadmium oxide/TEMPO-oxidized cellulose nanocomposites produced by pulsed laser ablation in liquid environment: Synthesis, characterization, and antimicrobial activity. Opt. Laser Technol..

[25] Mostafa A.M., Mwafy E.A., Hasanin M.S. (2020). One-pot synthesis of nanostructured CdS, CuS, and SnS by pulsed laser ablation in liquid environment and their antimicrobial activity. Opt. Laser Technol..

[26] Mostafa A.M., Mwafy E.A. (2020). Synthesis of ZnO and Au@ZnO core/shell nano-catalysts by pulsed laser ablation in different liquid media. J. Mater. Sci. Technol..

[27] Altowyan A.S., Mostafa A.M., Ahmed H.A. (2021). Effect of liquid media and laser energy on the preparation of Ag nanoparticles and their nanocomposites with Au nanoparticles via laser ablation for optoelectronic applications. Optik.

[28] Mostafa A.M., Mwafy E.A., Awwad N.S., Ibrahium H.A. (2021). Au@Ag core/shell nanoparticles prepared by laser-assisted method for optical limiting applications. J. Mater. Sci. Mater. Electron..

[29] Mukwevho N., Gusain R., Fosso-Kankeu E., Kumar N., Waanders F., Ray S.S. (2020). Removal of naphthalene from simulated wastewater through adsorption-photodegradation by ZnO/Ag/GO nanocomposite. J. Ind. Eng. Chem..

[30] Abbas S., Nasreen S., Haroon A., Ashraf M.A. (2020). Synhesis of Silver and Copper Nanoparticles from Plants and Application as Adsorbents for Naphthalene decontamination. Saudi J. Biol. Sci..

[31] Sudheeshkumar V., Sulaiman K.O., Scott R.W.J. (2020). Activation of atom-precise clusters for catalysis. Nanoscale Adv..

[32] Sulaiman K.O., Sudheeshkumar V., Scott R.W.J.R.a. (2019). Activation of atomically precise silver clusters on carbon supports for styrene oxidation reactions. RSC Adv..

[33] Mwafy E.A. (2020). Eco-friendly approach for the synthesis of MWCNTs from waste tires via chemical vapor deposition. Environ. Nanotechnol. Monit. Manag..

[34] Talaber I., van Gestel C.A., Kokalj A.J., Marolt G., Novak S., Zidar P., Drobne D. (2020). Comparative biokinetics of pristine and sulfidized Ag nanoparticles in two arthropod species exposed to different field soils. Environ. Sci. Nano.

[35] Wang J.-X., Wen L.-X., Wang Z.-H., Chen J. (2006). Physics, Immobilization of silver on hollow silica nanospheres and nanotubes and their antibacterial effects. Mater. Chem. Phys..

[36] Sarkar S., Das R.J.M., Letters N. (2018). Shape effect on the elastic properties of Ag nanocrystals. Micro Nano Lett..

[37] ElFaham M.M., Mostafa A.M., Mwafy E.A. (2021). The effect of reaction temperature on structural, optical and electrical properties of tunable ZnO nanoparticles synthesized by hydrothermal method. J. Phys. Chem. Solids.

[38] Mostafa A.M. (2021). Preparation and study of nonlinear response of embedding ZnO nanoparticles in PVA thin film by pulsed laser ablation. J. Mol. Struct..

[39] Li C., Fan X., Yu L., Cui L., Yin M., Li Y., Nan N., Liu N. (2020). A resistive-type UV detector based on ZnO nanowalls decoated by Ag nanowires. Opt. Mater..

[40] Ferreira E., Kharisov B., Vázquez A., Méndez E.A., Severiano-Carrillo I., Trejo-Durán M. (2020). Tuning the nonlinear optical properties of Au@ Ag bimetallic nanoparticles. J. Mol. Liq..

[41] Feng Y., Yin J., Liu S., Wang Y., Li B., Jiao T. (2020). Facile Synthesis of Ag/Pd Nanoparticle-Loaded Poly(ethylene imine) Composite Hydrogels with Highly Efficient Catalytic Reduction of 4-Nitrophenol. ACS Omega.

[42] Bhardwaj S., Sharma D., Kumari P., Pal B. (2020). Influence of photodeposition time and loading amount of Ag co-catalyst on growth, distribution and photocatalytic properties of Ag@TiO2 nanocatalysts. Opt. Mater..

[43] Mostafa A.M., Mwafy E.A., Lotfy V.F., Basta A.H. (2019). Optical, electrical and mechanical studies of paper sheets coated by metals (Cu and Ag) via pulsed laser deposition. J. Mol. Struct..

[44] Mostafa A.M., Lotfy V.F., Mwafy E.A., Basta A.H. (2020). Influence of coating by Cu and Ag nanoparticles via pulsed laser deposition technique on optical, electrical and mechanical properties of cellulose paper. J. Mol. Struct..

[45] Ahmad-Fouad Basha M., Mostafa A.M. (2019). UV-induced macromolecular and optical modifications in gelatin solid films with transition metal chlorides. J. Mol. Struct..

[46] Alghool S., Abd El-Halim H.F., Mostafa A.M. (2019). An Eco-friendly Synthesis of V2O5 Nanoparticles and Their Catalytic Activity for the Degradation of 4-Nitrophrnol. J. Inorg. Organomet. Polym. Mater..

[47] Pimentel A., Araújo A., Coelho B., Nunes D., Oliveira M., Mendes M., Águas H., Martins R., Fortunato E. (2017). 3D ZnO/Ag surface-enhanced Raman scattering on disposable and flexible cardboard platforms. Materials.

[48] Dixit S., Singhal S., Vankar V.D., Shukla A.K. (2017). Size dependent Raman and absorption studies of single walled carbon nanotubes synthesized by pulse laser deposition at room temperature. Opt. Mater..

[49] Hareesh K., Joshi R.P., Sunitha D.V., Bhoraskar V.N., Dhole S.D. (2016). Anchoring of Ag-Au alloy nanoparticles on reduced graphene oxide sheets for the reduction of 4-nitrophenol. Appl. Surf. Sci..

[50] Balachandran S., Praveen S.G., Velmurugan R., Swaminathan M. (2014). Facile fabrication of highly efficient, reusable heterostructured Ag–ZnO–CdO and its twin applications of dye degradation under natural sunlight and self-cleaning. RSC Adv..

[51] Das S., Alford T. (2013). Structural and optical properties of Ag-doped copper oxide thin films on polyethylene napthalate substrate prepared by low temperature microwave annealing. J. Appl. Phys..

[52] Tom R.T., Nair A.S., Singh N., Aslam M., Nagendra C., Philip R., Vijayamohanan K., Pradeep T.J.L. (2003). Freely dispersible Au@ TiO_2_, Au@ ZrO_2_, Ag@ TiO_2_, and Ag@ ZrO_2_ core− shell nanoparticles: One-step synthesis, characterization, spectroscopy, and optical limiting properties. Langmuir.

[53] Omrani N., Nezamzadeh-Ejhieh A. (2020). A comprehensive study on the mechanism pathways and scavenging agents in the photocatalytic activity of BiVO_4_/WO_3_ nano-composite. J. Water Process. Eng..

[54] Altowyan A.S., Ahmed H.A., Gomha S.M., Mostafa A.M. (2021). Optical and Thermal Investigations of New Schiff Base/Ester Systems in Pure and Mixed States. Polymers.

[55] Darwish W.M., Darwish A.M., Al-Ashkar E.A. (2016). Indium (III) phthalocyanine eka-conjugated polymer as high-performance optical limiter upon nanosecond laser irradiation. High Perform. Polym..

[56] El-Saied H., Mostafa A.M., Hasanin M.S., Mwafy E.A., Mohammed A.A. (2020). Synthesis of antimicrobial cellulosic derivative and its catalytic activity. J. King Saud Univ. Sci..

[57] Alamro F.S., Ahmed H.A., Naoum M.M., Mostafa A.M., Alserehi A.A. (2021). Induced Smectic Phases from Supramolecular H-Bonded Complexes Based on Non-Mesomorphic Components. Crystals.

[58] Arizavi A., Mirbagheri N.S., Hosseini Z., Chen P., Sabbaghi S. (2020). Efficient removal of naphthalene from aqueous solutions using a nanoporous kaolin/Fe_3_O_4_ composite. Int. J. Environ. Sci. Technol..

[59] Zeng G., You H., Du M., Zhang Y., Ding Y., Xu C., Liu B., Chen B., Pan X. (2021). Enhancement of photocatalytic activity of TiO2 by immobilization on activated carbon for degradation of aquatic naphthalene under sunlight irradiation. Chem. Eng. J..

[60] Gao Y., Li S., Zhao B., Zhai Q., Lita A., Dalal N.S., Kroto H.W., Acquah S.F.A. (2014). A synergistic approach to light-free catalysis using zinc oxide embedded multi-walled carbon nanotube paper. Carbon.

[61] Giovannetti R., Rommozzi E., D’Amato C.A., Zannotti M.J.C. (2016). Kinetic model for simultaneous adsorption/photodegradation process of alizarin red S in water solution by nano-TiO_2_ under visible light. Catalysts.

[62] Chen C.-Y., Hsu L.-J. (2015). Kinetic study of self-assembly of Ni (II)-doped TiO_2_ nanocatalysts for the photodegradation of azo pollutants. RSC Adv..

[63] Mohamed M.M., Osman G., Khairou K.S. (2015). Fabrication of Ag nanoparticles modified TiO_2_–CNT heterostructures for enhanced visible light photocatalytic degradation of organic pollutants and bacteria. J. Environ. Chem. Eng..

[64] Yan Y., Sun H., Yao P., Kang S.-Z., Mu J. (2011). Effect of multi-walled carbon nanotubes loaded with Ag nanoparticles on the photocatalytic degradation of rhodamine B under visible light irradiation. Appl. Surf. Sci..

[65] Liu D., Wu Z., Tian F., Ye B.-C., Tong Y. (2016). Compounds, Synthesis of N and La co-doped TiO_2_/AC photocatalyst by microwave irradiation for the photocatalytic degradation of naphthalene. J. Alloy. Compd..

